# A Sensitive Fluorescence Biosensor for Silver Ions (Ag^+^) Detection Based on C-Ag^+^-C Structure and Exonuclease III-Assisted Dual-Recycling Amplification

**DOI:** 10.1155/2019/3712032

**Published:** 2019-03-03

**Authors:** Yubin Li, Jiaming Yuan, Zexi Xu

**Affiliations:** ^1^School of Chemistry and Environment, Guangdong Ocean University, Zhanjiang 524088, China; ^2^School of Food Science and Nutrition, University of Leeds, Leeds LS2 9JT, UK

## Abstract

A C-Ag^+^-C structure-based fluorescence biosensor with novel combination design of exonuclease III (Exo III) dual-recycling amplification is proposed for the application of silver ions (Ag^+^) detection. Since oligo-1 involves C-C mismatches, the presence of Ag^+^ can be captured to form C-Ag^+^-C base pairs, which results in a double-helix structure with a blunt terminus. The double-helix structure can be cleaved by EXO III to release short mononucleotide fragments (trigger DNA) and Ag^+^. Released Ag^+^ can form new bindings with oligo-1, and other trigger DNA can be produced in the digestion cycles. Hybridization with the signal DNA (oligo-2) transforms a trigger DNA into double-stranded DNA with blunt terminus which can be cleaved by Exo III to reproduce the trigger DNA and form guanine- (G-) quadruplex DNA. The trigger DNA returns free to the solution and hybridizes with another signal DNA, which realizes the dual-recycling amplification. The G-quadruplex DNA can be reported by *N*-methylmesoporphyrin IX (NMM), a specific G-quadruplex DNA fluorochrome. This method allows Ag^+^ to be determined in the 5 to 1500 pmol/L concentration range, with a 2 pmol/L detection limit, and it has been successfully applied to the detection of Ag^+^ in real samples.

## 1. Introduction

Silver is the oldest known metal and widely used in photography, pharmacy, and semiconductor industry [1, 2]. However, silver ions (Ag^+^) are one of the most hazardous metal pollutants that can be widely distributed in air, water, soil, and even food [3–6]. Furthermore, due to the interaction with various metabolites and inactivating sulfhydryl enzymes, Ag^+^ can produce dose-dependent cytopathogenic effects in many types of cells including human gingival fibroblast, keratinocytes, human tissue mast cell, and endothelial cell [7–9]. Therefore, it is of great importance to develop a sensitive and selective method for Ag^+^ detection in environmental, biomedical, food, and other related samples. Traditional methods for detecting trace amounts of Ag^+^ include atomic absorption spectrometry (AAS) [10, 11], inductively coupled plasma-mass spectrometry (ICP-MS) [12–14], and ionic selective electrode (ISE) [15]. However, the expensive instrumentation, the complex sample preparations, and the need for skilled technicians limit the applications of these traditional methods [16, 17]. Thus, the development of rapid and simple methods for Ag^+^ detection is an urgent need.

Recently, the high specificity of the interaction of nucleic acid bases with metal ions has been successfully used in the study of heavy metal ion detection. Ono et al. reported that Ag^+^ could interact with cytosine (C) base selectively to form a stable C-Ag^+^-C structure through coordinate bonds, which transforms the single-stranded DNA into a double-helix structure. Also, the specific C-Ag^+^-C interaction guarantees excellent selectivity because the C-C mismatches have interaction with Ag^+^ instead of other metal ions [18, 19]. Compared with various Ag^+^ biosensors applying this principle such as colorimetric biosensors [20, 21] and electrochemical biosensors [22, 23], fluorescent biosensors have attracted tremendous attention owing to their operational convenience, less time-consumption, and high sensitivity [5, 24].

These methods based on combining metal ions analysis with enzymatic amplification strategies have been developed and recognized as a powerful tool to improve the sensitivity for metal ions detection [25–27], especially the enzymatic amplification strategies based on Exo III, presenting simple and sensitive detection of metal ions [27, 28]. Compared with other nicking endonuclease, which requires specific recognition sites, Exo III can selectively catalyze the stepwise removal of mononucleotides from the blunt or the recessed 3′-termini of double-stranded DNA (DNA hairpin) without any requirement of specific recognition sites, and this action is limited while 3′-overhang ends of double-stranded DNA or single-stranded DNA [29, 30]. Therefore, Exo III provides huge value for amplified metal ions detection.

In this study, we demonstrate a homogeneous fluorescence biosensor for Ag^+^ detection using the C-Ag^+^-C structure with signal amplification by Exo III-assisted dual-recycling. Based on the C-Ag^+^-C structure, Ag^+^ can be captured by oligo-1 to form the DNA hairpin with blunt terminus which can be digested by Exo III, producing short mononucleotide fragment (trigger DNA) and releasing the Ag^+^ for a new cycle. Similarly, based on the Exo III-assisted amplification, released trigger DNA repeatedly open oligo-2 and promote the generation of plentiful G-quadruplex DNA. *N*-Methylmesoporphyrin IX (NMM) is selected as the fluorochrome because its fluorescence intensity presented apparent enhancement, owing to the formation of the G-quadruplex-NMM complex [31, 32]. The proposed method displays high distinction efficiency towards Ag^+^ against other environmentally relevant metal ions, and it is successfully applied to the detection of Ag^+^ in real samples.

## 2. Experimental Section

### 2.1. Reagents

Oligo-1 and oligo-2 are the sequences of capture DNA and signal DNA, respectively. The oligo-1 and oligo-2 were separately heated at 90°C for 10 min and then slowly cooled to room temperature to form the satisfactory hairpin loop. All oligonucleotides were synthesized and purified by Sangon Bioengineering Technology and Services Co. Ltd. (Shanghai, China). Exo III was purchased from the Thermo Fisher Scientific Inc. (USA). Tris, potassium nitrate, magnesium nitrate, and silver nitrate were purchased from Sinopharm Chemical Reagent Co. Ltd. (Beijing, China). *N*-Methylmesoporphyrin IX (NMM) was purchased from Lark technology Co. Ltd. (Beijing, China). All reagents used were of analytical reagent grade. Nanopure water (18.1 MΩ) was obtained from a 350 Nanopure water system (Guangzhou Crystalline Resource Desalination of Sea Water and Treatment Co. Ltd.) and was used in all experiments. All oligonucleotides stock solutions were prepared by dissolving oligonucleotides in the Tris-HNO_3_ buffer (50 mmol/L, pH 7.0, 100 mmol/L NaNO_3_, and 5 mmol/L Mg(NO_3_)_2_). All the solutions were stored at 4°C before using.

### 2.2. Apparatus and Conditions

The value of pH was measured by using a pH meter (pHS-3E, Shanghai Lei-ci Instrument Plant, China). Fluorescence was measured by F-4600 Hitachi fluorescence spectrometer (Japan) equipped with a xenon lamp excitation source under 25°C using a quartz cell of 1 cm path length. The excitation wavelength was 399 nm, and the emission spectra were collected from 580 to 650 nm. The fluorescence intensity at 614 nm was used to choose the optimal experimental conditions and evaluate the performance of the sensing system. The excitation and emission slit widths were both set at 5 nm.

### 2.3. Sensor Fabrication

For the sensing procedure, 10 nmol/L oligo-1, 40 nmol/L oligo-2, 10 U Exo III, and different concentrations of Ag^+^ (buffer for blank) were incubated at 37°C for 50 min. Then, 50 mmol/L K^+^, 160 nmol/L NMM, and Tris-HNO_3_ buffer solution were added. The final solution with a total volume of 500 *μ*L was incubated at 37°C for 10 min followed by the fluorescence measurement.

### 2.4. Real Sample Measurements

Fluorescence spectrometry was applied to detect Ag^+^ in Huguang Lake water of Zhanjiang City and human sera to verify the feasibility of this method. The impurities in the water sample were filtered through a filter membrane with the pore size of 0.45 *μ*m. Next, aliquots of the lake water samples or human sera samples were spiked with a stock solution of Ag^+^ and diluted 5 times with higher concentration of Tris-HNO_3_ buffer. The Ag^+^ fluorescence measurement was then performed in the same method.

## 3. Results and Discussion

### 3.1. Principle of the Biosensor

**Figure 1:**
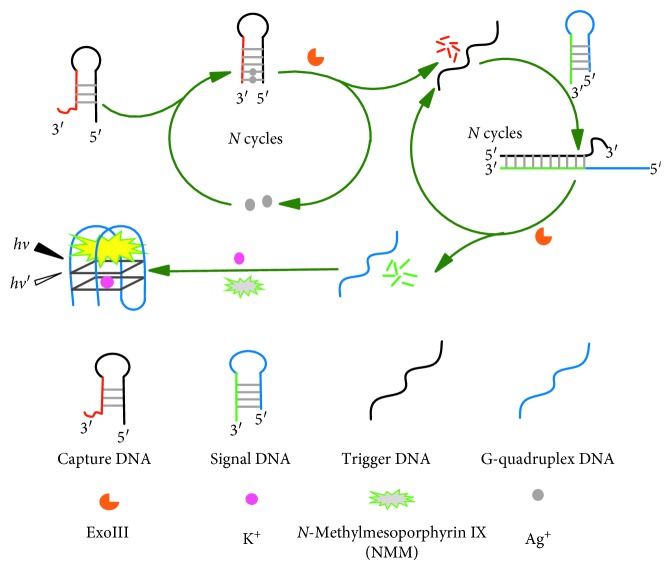
Scheme for detection of Ag^+^ with the dual-recycling amplification of exonuclease III.

The fluorescence biosensor of Ag^+^ is constructed based on the C-Ag^+^-C structure and Exo III-assisted dual-recycling amplification, and relevant experimental principle is illustrated in Figure 1. The hairpin probe 5′-A**CC C**AA ATG GGT AGG GCT AGGC GCC CTA CCC ATT T**CC C**T-3′ is designed as capture DNA (oligo-1), the underlined sequence is the stem region, and there are three mismatches of C-C bases at 5′ termini and 3′ termini (bold sequence). In the absence of Ag^+^, Exo III has no cleavage activity on 3′-protruding termini, and oligo-1 cannot be digested by Exo III. However, oligo-1 can capture Ag^+^ through C-C mismatches to form C-Ag^+^-C base pairs with the presence of Ag^+^. The C-Ag^+^-C interaction transforms the stem region of oligo-1 into a double-helix structure with blunt terminus. Then, Exo III selectively digests the double-helix structure in the direction of 3′ to 5′, and Ag^+^ is released for new binding with the other oligo-1. With each digestion cycle, a digestion product (5′-ACC CAA ATG GGT AGG GCT AGG C-3′, trigger DNA) is obtained. The trigger DNA can open the hairpin probe 5′-**TGG GTA GGG CGG GTT GGG** TTT TTT GCC CTA CCC ATT TGG GT-3′ (signal DNA, oligo-2) to form DNA hairpin with blunt 3′-termini which can be cleaved by Exo III, producing the G-rich fragment (bold sequence) locked by the underlined sequence before digestion. The released trigger DNA can hybridize with another signal DNA, which realizes the dual-recycling amplification, and the free G-rich fragment in oligo-2 can form G-quadruplex-NMM complex with fluorescence enhancement. Thus, the concentration of Ag^+^ could be easily monitored by observing fluorescence changes of this sensing system.

### 3.2. Feasibility of the Biosensor

**Figure 2:**
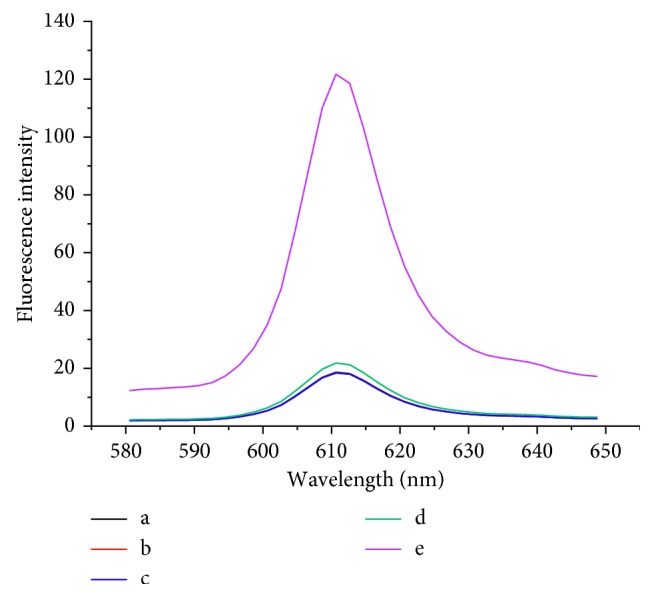
Fluorescence emission spectra of different solutions: (a) 10 nmol/L oligo-1 + 1500 pmol/L Ag^+^; (b) 40 nmol/L oligo-2 + 1500 pmol/L Ag^+^; (c) 10 nmol/L oligo-1 + 40 nmol/L oligo-2; (d) 10 nmol/L oligo-1 + 40 nmol/L oligo-2 + 10 U Exo III; (e) 10 nmol/L oligo-1 + 40 nmol/L oligo-2 + 10 U Exo III + 1500 pmol/L Ag^+^. Experimental conditions: 50 mmol/L K^+^ and 160 nmol/L NMM.

To investigate the possibility of the assay, fluorescence emission spectra of different mixtures were recorded.  Figure 2 shows a low signal when it is capture DNA with Ag^+^ (curve a). Similarly, the fluorescence signal of signal DNA with Ag^+^ (curve b) or capture DNA with signal DNA (curve c) is almost the same as that of curve a due to the lack of G-rich sequence; therefore, it is difficult to distinguish curve a, b, and c in Figure 2. The fluorescence intensity had only a slight increase (curve d) with the introduction of Exo III, which means the G-rich sequence cannot be released efficiently in the absence of Ag^+^. However, with the addition of Ag^+^, the fluorescence intensity is increased obviously (curve e), because the Ag^+^ and capture DNA hybridize based on the formation of the C-Ag^+^-C structure. Under the cleavage of Exo III, a number of trigger DNA are produced. These trigger DNA can hybridize with oligo-2 to form DNA hairpin with blunt terminus, which can be digested by Exo III to release G-rich fragment, resulting in strong signals. The results above strongly prove the feasibility of the proposed method for Ag^+^ detection.

### 3.3. Optimization of the Experimental Condition

To maximize the response signal of the proposed biosensor, various conditions were optimized. A key concept in our work is the cutting effect of EXO III and the formation of the G-quadruplex-NMM complex. Therefore, the following factors: the incubation time and temperature of Exo III, the concentration of Exo III and signal DNA, the signal DNA/capture DNA ratio, and pH value were optimized to control the fluorescence signal. Their effects on the change of fluorescence intensity (Δ*I*) were estimated through the equation Δ*I* = *I*_target_ − *I*_blank_, where *I*_blank_ represents the fluorescence intensity of the mixture of oligo-1, oligo-2, and Exo III and *I*_target_ denotes the fluorescence intensity of the mixture of oligo-1, oligo-2, and Exo III in the presence of 1500 pmol/L Ag^+^.

**Figure 3:**
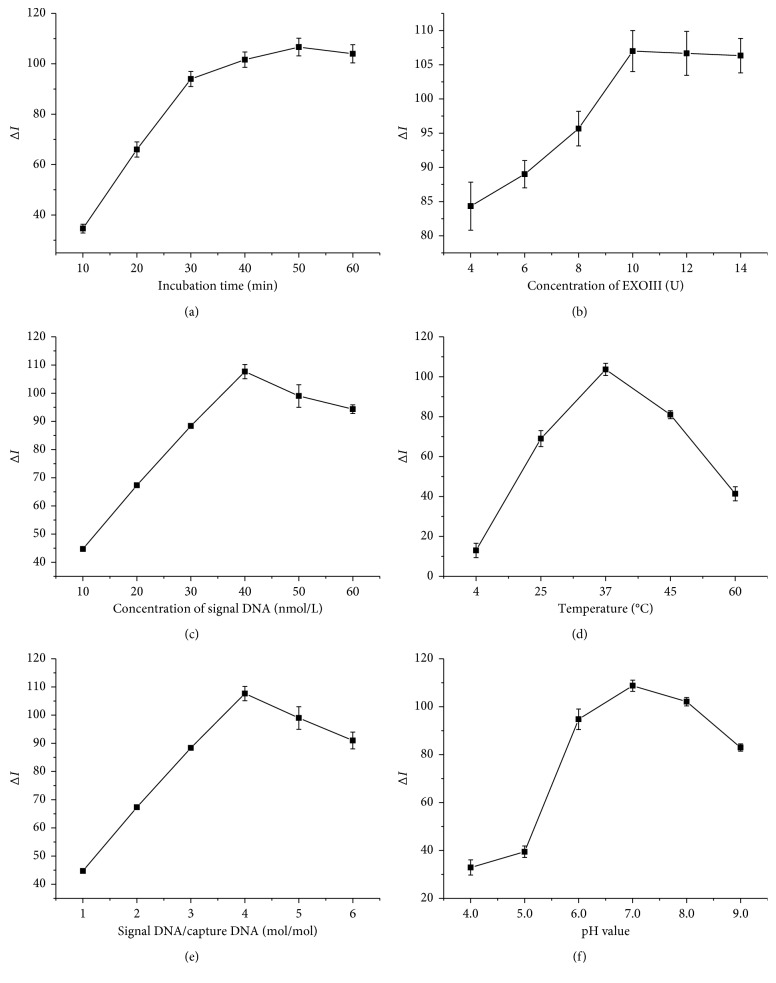
Optimization of experimental conditions: (a) effect of the incubation time of Exo III digestion; (b) effect of Exo III concentration; (c) effect of signal DNA concentration; (d) effect of the incubation temperature; (e) effect of the signal DNA/capture DNA ratio; (f) effect of the pH value. Experimental conditions: 10 nmol/L oligo-1, 40 nmol/L oligo-2, 10 U Exo III, 50 mmol/L K^+^, and 160 nmol/L NMM. Error bars represent the standard deviation of three independent experiments.

As shown in Figure 3(a), Δ*I* reaches a maximum when the incubation time is 50 min and remains relatively stable in longer reaction time. This result indicated that further incubation time (more than 50 min) was not necessary for the cycles. As displayed in Figure 3(b), Δ*I* reaches the maximum and remains a steady value when the amount of Exo III increases to 10 U. Thus, the amount of Exo III was set at 10 U for subsequent experiments. Furthermore, the concentration of signal DNA has been explored. A strong background signal is an unavoidable result of high concentration of signal DNA, and the optimal concentration of signal DNA was set as 40 nM according to Figure 3(c). As displayed in Figure 3(d), experiments showed Exo III had a much better activity at a temperature of 37°C. Therefore, all the experiments were conducted at 37°C. In addition, Δ*I* reached a maximum when the signal DNA/capture DNA ratio was 4 and decreased along with the ratio exceeding 4 Figure 3(e). Hence, 4 was chosen as the optimal signal DNA/capture DNA ratio in the following experiments. Ultimately, as demonstrated in Figure 3(f), Δ*I* reaches a maximum when the pH = 7.0. Consequently, the optimal value of pH was set as 7.0.

### 3.4. Sensitivity of the Biosensor

**Figure 4:**
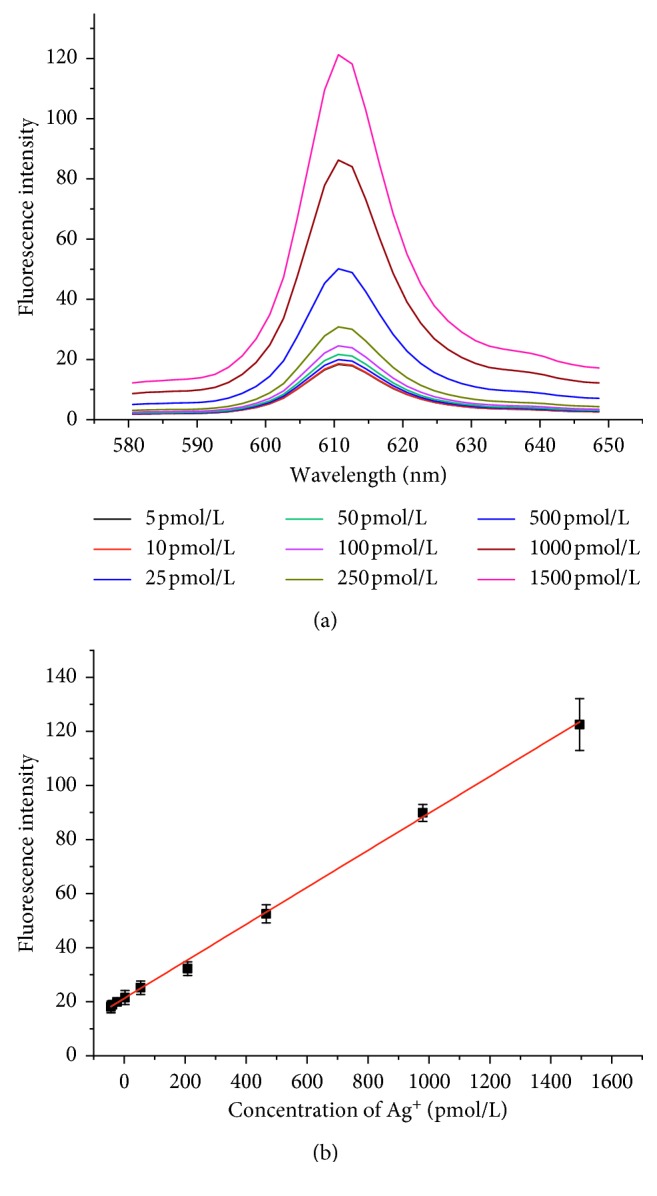
Fluorescence spectra (a) and calibration plot (b) for Ag^+^. Experimental conditions: 10 nmol/L oligo-1, 40 nmol/L oligo-2, 10 U Exo III, 50 mmol/L K^+^, 160 nmol/L NMM, and 50 min incubation time. Error bars represent the standard deviation of three independent experiments.

The sensitivity of the biosensor was investigated with different concentrations of Ag^+^ under optimum conditions. As shown in Figure 4, the fluorescence intensity increases with the increasing Ag^+^ concentration. The intensity is proportional to the concentration of Ag^+^ over the range from 5 pmol/L to 1500 pmol/L, with a linear regression equation of *I* = 0.07°*C* + 17.48 (*C*: pmol/L, *R* = 0.998, *C* represents the concentration of Ag^+^, and *I* denotes the fluorescence intensity) and a detection limit of 2 pmol/L, which is obtained from the equation of DL = 3*б*/slope (*б*: relative standard deviation of the blank sample; slope: the slope of linear regression equation).

The comparisons with other methods are listed in Table 1. The detection limit of our proposed assay is considerably satisfactory, which is lower than those of some reported methods. Therefore, the proposed biosensor for Ag^+^ detection represents a promising practical application.

### 3.5. Selectivity, Reproducibility, and Stability of the Biosensor

**Figure 5:**
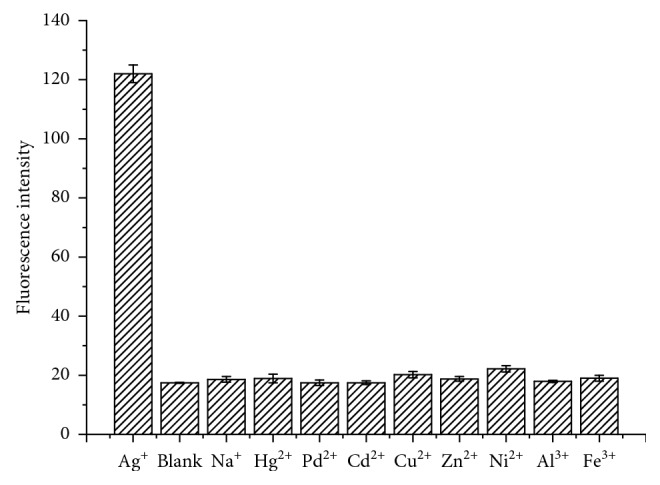
Selectivity of the biosensor. Experimental conditions: 10 nmol/L oligo-1, 40 nmol/L oligo-2, 10 U Exo III, 50 mmol/L K^+^, 160 nmol/L NMM, and 50 min incubation time. The concentration of Ag^+^ was 1.5 nmol/L, and the concentrations of other metal ions were 15 nmol/L. Error bars represent the standard deviation of three independent experiments.

To evaluate the ion selectivity of the proposed biosensor, an assay was applied by using other environmentally relevant metal ions including Na^+^, Hg^2+^, Pd^2+^, Cd^2+^, Cu^2+^, Zn^2+^, Ni^2+^, Al^3+^, and Fe^3+^ (15 nmol/L), and the responses were investigated under the same conditions as the case of Ag^+^. According to Figure 5, compared with other metal ions, the addition of Ag^+^ (1.5 nmol/L) has a remarkable change in the fluorescence signal. This result reveals that Ag^+^ can specifically interact with C-C mismatches to form strong and stable C-Ag^+^-C structures, indicating that the sensing system provides excellent specificity and selectivity for the detection of Ag^+^ against other interfering metal ions at higher concentrations.

To estimate the reproducibility of the proposed biosensor, nine repetitive measurements on 1.5 nmol/L Ag^+^ were performed and yielded reproducible response current signals with a relative standard deviation of 0.7%. The stability of the Ag^+^ sensing assay was also investigated by repetitive experiments. The result indicated that the fluorescence intensity decreased <6.5% (RSD = 1.2%) after 10 continuous scans. All these experimental results showed that this biosensor has a very good reproducibility and stability.

### 3.6. Ag^+^ Detection in Real Samples

The application of the proposed biosensor in real samples was investigated by measuring the recovery of spiked Ag^+^ in Huguang lake water (Table 2) and human sera (Table 3). The recovery was in the range of 99.5–105.8% for four Huguang lake water samples with relative standard deviation (RSD) of 1.9–4.8%. For the human sera, the respective data were 96.6–108.0% and 3.1–6.1%. The results were then compared with those obtained through atomic absorption spectrometry (AAS), which served as a reference standard. The promising values of recovery and RSD confirm fine reliability and great practicability of the proposed biosensor for Ag^+^ detection in real samples.

## 4. Conclusions

In this work, we have developed a simple, sensitive, selective and homogeneous fluorescence biosensor for the Ag^+^ detection based on the C-Ag^+^-C structure and Exo III-assisted dual-recycling amplification. There are several significant advantages of the proposed biosensor. Firstly, the developed method is highly selective because of the C-Ag^+^-C coordination chemistry, which enables accurate detection of Ag^+^ in the presence of other metal ions. Besides, the developed method greatly lowers the detectable limit towards Ag^+^ down to 2 pmol/L due to Exo III-assisted dual-recycling amplification. Moreover, the proposed biosensor has excellent reproducibility for Ag^+^ detection. Therefore, we believe that the developed analytical method has great potential applications in the Ag^+^ detection in real environmental, biomedical, and other samples.

## Figures and Tables

**Table 1:** Comparison of the proposed assay with some reported methods.

Signal amplification strategy	Linear range	Detection limit	Apply to real samples	Reference
Quartz crystal microbalance (QCM) and silver-specific DNAs	100 pmol/L–1 *μ*mol/L	100 pmol/L	Yes	[33]
Single-layer MoS_2_ nanosheets	1 nmol/L–100 nmol/L	1 nmol/L	Yes	[34]
Nanoporous gold/anionic intercalator	0.1 nmol/L–1 *μ*mol/L	0.48 pmol/L	Yes	[35]
Cationic polymer-directed AuNPs aggregation	80 nmol/L–10000 nmol/L	48.6 nmol/L	No	[36]
Hemin/G-quadruplex nanowire	0.1 nmol/L–100 mmol/L	0.05 nmol/L	Yes	[37]
Nanographite-DNA hybrid and DNase I	1 nmol/L–200 nmol/L	0.3 nmol/L	Yes	[38]
HAC-DNA interaction	0.1 *μ*mol/L–75 *μ*mol/L	58 nmol/L	No	[39]
C-Ag^+^-C structure and exonuclease III	5 pmol/L–1500 pmol/L	2 pmol/L	Yes	This work

**Table 2:** Determination of Ag^+^ in Huguang lake water samples (*n*=3).

Samples	Added (pmol/)	Proposed method (mean ± SD) (pmol/L)	AAS (mean ± SD) (pmol/L)
1	5.0	5.1 ± 0.3	4.8 ± 0.1
2	50.0	52.9 ± 1.8	49.1 ± 1.4
3	500.0	506.7 ± 12.0	503.4 ± 16.3
4	1500.0	1493.4 ± 28.5	1520.1 ± 34.5

Mean represents the average of three determinations; SD, standard deviation.

**Table 3:** Determination of Ag^+^ in human sera samples (*n*=3).

Samples	Added (pmol/)	Proposed method (mean ± SD) (pmol/L)	AAS (mean ± SD) (pmol/L)
1	5.0	5.4 ± 0.2	5.1 ± 0.1
2	50.0	48.3 ± 1.6	49.3 ± 1.7
3	500.0	511.2 ± 13.4	506.9 ± 11.0
4	1500.0	1481.2 ± 26.8	1543.6 ± 21.3

Mean represents the average of three determinations; SD, standard deviation.

## Data Availability

The data used to support the findings of this study are included within the article. And readers can access the data supporting the conclusions of the study from our manuscript.
